# LarynxFormer: a transformer-based framework for processing and segmenting laryngeal images

**DOI:** 10.3389/fdgth.2025.1459136

**Published:** 2025-07-11

**Authors:** Rune Mæstad, Abdul Hanan, Haakon Kristian Kvidaland, Hege Clemm, Reza Arghandeh

**Affiliations:** ^1^ Faculty of Engineering and Science, Western Norway University of Applied Sciences, Bergen, Vestland, Norway; ^2^ Faculty of Health and Social Sciences, Western Norway University of Applied Sciences, Bergen, Vestland, Norway; ^3^ Department of Pediatric and Adolescent Medicine, Haukeland University Hospital, Bergen, Vestland, Norway; ^4^ Department of Clinical Science, University of Bergen, Bergen, Vestland, Norway; ^5^ Department of Head and Neck, Haukeland University Hospital, Bergen, Vestland, Norway

**Keywords:** exercise-induced laryngeal obstruction, continuous laryngoscopy exercise test, machine learning, artificial intelligence, image segmentation

## Abstract

Manual diagnostic methods for assessing exercise-induced laryngeal obstruction (EILO) contain human bias and can lead to subjective decisions. Several studies have proposed machine learning methods for segmenting laryngeal structures to automate and make diagnostic outcomes more objective. Four state-of-the-art models for laryngeal image segmentation are implemented, trained, and compared using our pre-processed dataset containing laryngeal images derived from continuous laryngoscopy exercise-test (CLE-test) data. These models include both convolutional-based and transformer-based methods. We propose a new framework called LarynxFormer, consisting of a pre-processing pipeline, transformer-based segmentation, and post-processing of laryngeal images. This study contributes to the investigation of using machine learning as a diagnostic tool for EILO. Furthermore, we show that a transformer-based approach for larynx segmentation outperforms conventional state-of-the-art image segmentation methods in terms of performance metrics and computational speed, demonstrating up to 2x faster inference time compared to the other methods.

## 1 Introduction

Exercise–induced laryngeal obstruction (EILO) is a condition where the laryngeal structures narrow during physical activity, resulting in significant breathing difficulties (1). The condition is more common among athletes and active youth (2), with a prevalence ranging from 5% to 8% (3–5). EILO affects exercise performance and quality of life and is often misdiagnosed as asthma (6).

Diagnosis of EILO is made using the continuous laryngoscopy exercise test (CLE-test), the gold standard within the field (7, 8). During the test, video recordings of the larynx are captured with a laryngoscope attached to a headset and inserted through the nose while the patient exercises on a treadmill or ergometer bike. The CLE score system (9) is employed to diagnose EILO but also to assess the severity of EILO at both glottic and/or supraglottic levels. Both levels are assigned a subscore between 0 and 3, where a higher score indicates more severe EILO. The CLE score is based on the relative degree of inspiratory adduction of glottic and supraglottic movements during exercise. EILO is present with a subscore equal to or above 2, and the sum of the subscores indicates the patient’s severity of EILO. However, The CLE score system has limitations, especially in terms of objectivity (10). The scores, determined by a doctor, contain a subjective bias, leading to potential inconsistency in the assessment.

Several studies have proposed machine learning (ML) models to identify laryngeal movements for more objective and consistent diagnostic methods. Each approach involves image segmentation on laryngeal images, aiming to automatically recognize key structures within the larynx, such as the trachea, vocal folds, and supraglottis. Referring to our previous work (11), we investigated relevant studies on laryngeal image segmentation.

Lin et al. (12) used convolutional networks to train a deep learning model to quantify and analyze the laryngeal closure. The researchers utilized a convolutional neural network (CNN) for region of interest detection and a fully convolutional network (FCN) for image segmentation of the laryngeal structures. A study by Choi et al. (13) trained a model with the Mask R-CNN architecture (14) to segment anatomical structures in laryngoscopy videos. Chen et al. developed the 3D VOSNet architecture for segmenting the larynx, with a focus on assessing muscle movement. The method stands out by its input, which takes a sequence of images, enabling a better understanding of the progression of the larynx movements. Fehling et al. (15) implemented a Convolutional Long Short-Term Memory (LSTM) network for segmenting the larynx’ glottal area in addition to the vocal folds on high-speed videos (HSV). Kruse et al. (16) proposed the GlottisNetV2 for “glottal midline detection using deep convolutional networks.” The network uses a U-Net architecture with convolutional layers.

Starting with Vaswant et al.’s paper on transformers (17), there has been significant progress in natural language processing. Following this, there has been an increasing interest in applying transformers to computer vision tasks. In 2021, Dosovitskiy et al. (18) introduced the Vision Transformer (ViT), achieving great results compared to state-of-the-art convolutional networks. Subsequently, the SegFormer architecture was proposed by Xie et al. (19), introducing a more simple and efficient method for image segmentation with transformers.

Utilizing the advancements in vision transformers, we introduce a framework called LarynxFormer to process and segment laryngeal images. The framework employs transformers for segmentation, utilizing the SegFormer architecture, which surpasses previous state-of-the-art methods. We compare this transformer-based approach with several other techniques, including FCN with a ResNet backbone, Mask R-CNN, and U-Net.

This explorative study aims to evaluate proposed state-of-the-art methods for laryngeal segmentation and compare them to more recent methods like transformers. It lays a foundation for future studies on utilizing ML to diagnose EILO and its contributions are:

• A transformer-based segmentation framework (LarynxFormer) specifically designed for laryngeal images, which has not been previously explored in the context of EILO. By comparing it to state-of-the-art architectures like U-Net, FCN, and Mask R-CNN, we highlight the potential advantages of transformer-based models in this domain. The framework also includes a pipeline for the pre-processing, segmentation, and post-processing of laryngeal images from CLE-tests.
• We implemented and evaluated the LarynxFormer framework for processing and analyzing EILO data provided by Bergen ILO Group.
• This work contributes to the exploration of using ML for future diagnosis methods, providing one of the initial steps for further investigation in this area.

This paper is structured into multiple sections: firstly, a data description of the raw data sourced from the Bergen ILO Group. Secondly, a methodology section explaining the proposed framework and its core components. The results and discussion section compares the performance of ML models across different metrics, inference times, and challenges. Lastly, conclusions and future research will be discussed.

## 2 Data description

**Figure 1:**
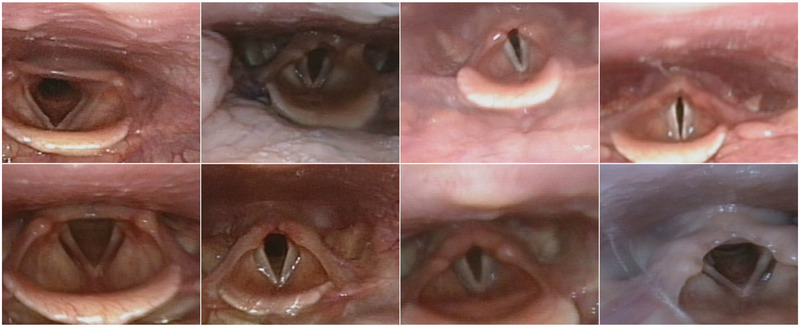
Examples of pre-processed laryngeal images from different patients. The top row shows an increasing level of narrowing at the glottic level, from left to right. The bottom row shows an increasing level of narrowing at the supraglottic level, from left to right.

The project used data from approximately 100 unique CLE-tests performed at Haukeland University Hospital. Each test comprises a recorded video lasting between 10 and 15 min. The anonymized videos contain recordings from multiple sources, including a screen recording from the cardiopulmonary exercise test (CPET) software tool and the patient’s larynx, recorded using a flexible fiberoptic laryngoscope (Olympus, Tokyo, Japan). Each recorded video has a resolution of 1920 × 1080 and a frame rate of 25 frames per second (FPS). Although the specific dimensions of the recording captured by the laryngoscope differ, the aspect ratio remains consistent. The videos selected for our dataset are the patients’ first attempts at the CLE-test, where the most intense EILO symptoms are expected to be present. Figure 1 shows examples of pre-processed laryngeal images with corresponding CLE-scores. The study and its use of patient data were approved by the Committee on Medical Research Ethics of Western Norway (REK numbers 2022-469975 and 2020-134444), and informed written consent was obtained from the participants.

## 3 Methodology

**Figure 2:**
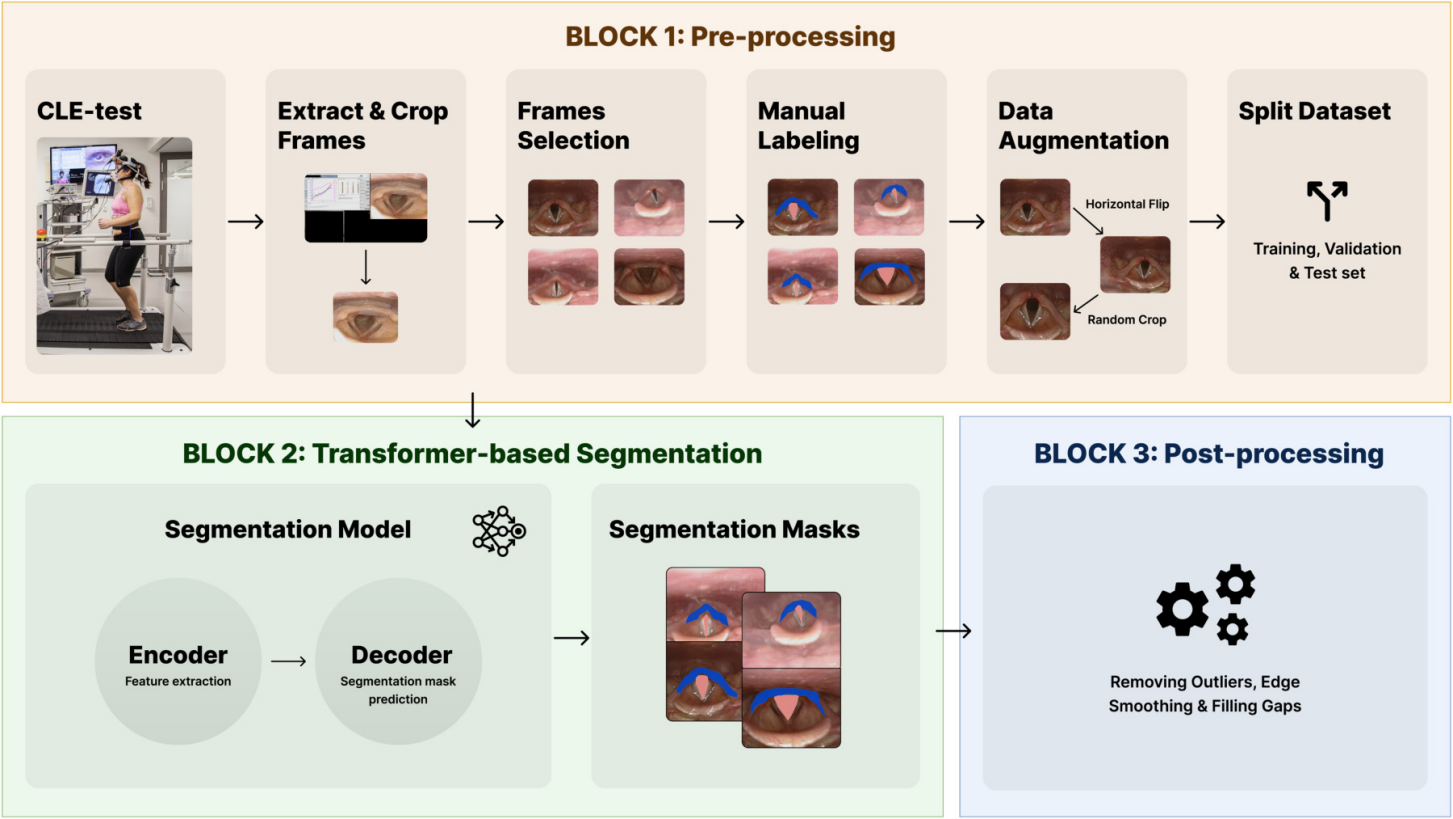
The blocks of the LarynxFormer framework. Block 1 shows the pre-processing steps. Block 2 is the segmentation part of the framework. Block 3 describes the post-processing steps. (Block 1, CLE-test image is reproduced with permission of the © ERS 2024: Eur Respir J 50(3) 1602221; DOI: 10.1183/13993003.02221-2016 Published 9 September 2017).

This section presents the LarynxFormer framework (Figure 2), slitted into a pre-processing, segmentation, and post-processing block.

### 3.1 Block 1: pre-processing

Several pre-processing steps were required before training the models. **CLE-test:** For each CLE-test video, it was necessary to divide it into frames and crop the section containing the larynx (**Extract and crop frames**). The position of the larynx recording differed for each video batch, requiring the use of an algorithm to calculate the crop boundaries. OpenCV (20), a Python package, was employed for all these tasks involving video processing.

Manual selection and labeling were required to capture clinically meaningful variation across obstruction levels, as automated methods could not reliably distinguish or annotate these specific conditions. **Frame selection:** Three cropped frames were selected from each CLE-test video. To ensure a balanced dataset, the selection process involved manually picking one frame with the larynx in a “normal” condition without obstruction, another with mild obstruction, and the final one showcasing the most severe symptoms. **Manual labeling:** Label Studio (21) was employed for labeling the laryngeal structures. To limit subjective bias and to ensure correct labeling, domain experts within EILO were involved in this step. The trachea and supraglottis were labeled, given their proven significant impact on EILO severity (9) and their essential role in determining the CLE-score. Using only two class labels, excluding the background, lowers the complexity of both the labeling and training procedure.

**Figure 3:**
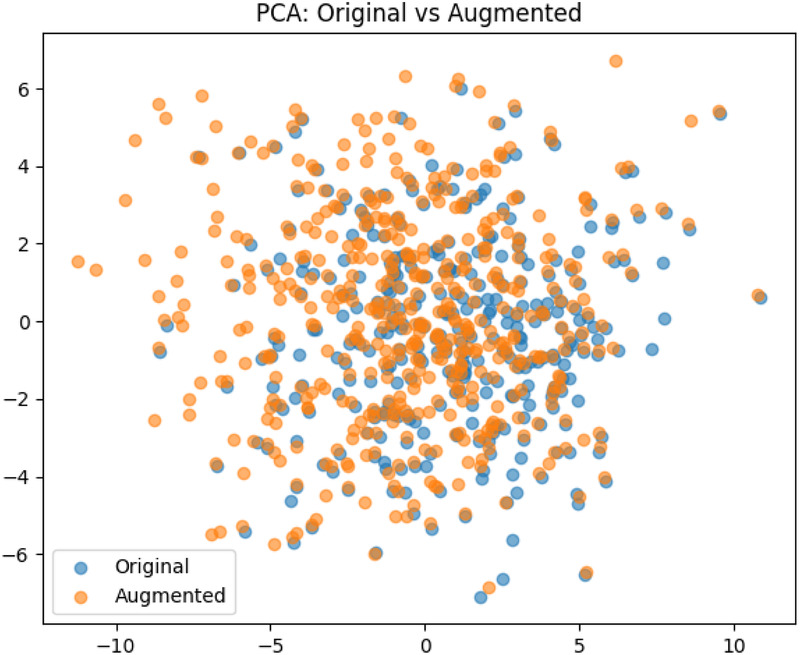
The result of the Principal Component Analysis on the original and augemented data used for training.

**Figure 4:**
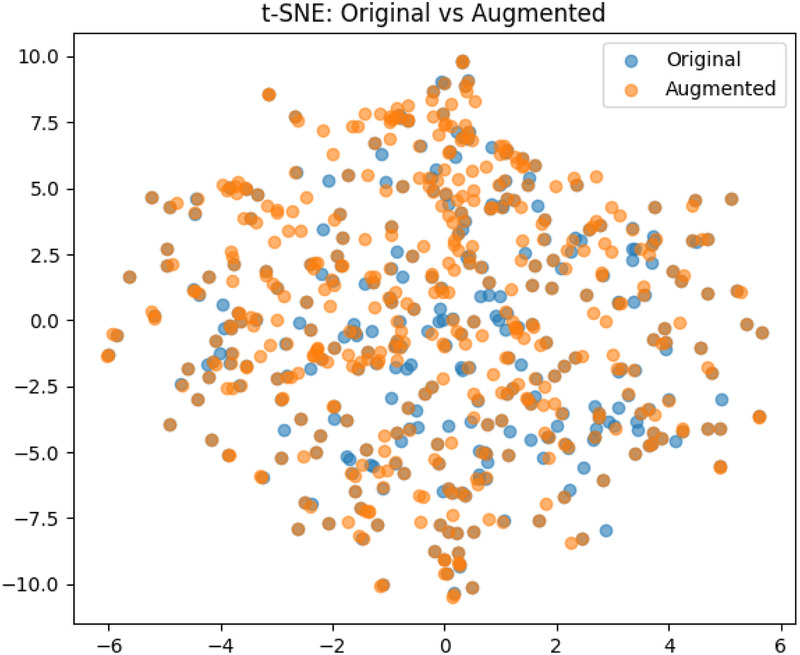
The result of the t-Distributed Stochastic Neighbor Embedding on the original and augemented data used for training.

The pre-processing step resulted in a dataset of 340 images. **Data augmentation:** To avoid overfitting of the models, and to enable better generalization, data augmentation techniques were applied. Utilizing the transformation package from PyTorch (22), we augmented the images by introducing random resizing crops and horizontal flips. These augmentation steps effectively doubled the size of our dataset. Embeddings were obtained from images to examine the feature-space relationship between original and augmented samples. Dimensionality reduction methods like PCA and t-SNE were applied to facilitate a clear comparison, independent of task-specific model inference. The visualizations (Figures 3, 4) reveal strong overlap between original and augmented data distributions, suggesting that augmentations have minimal impact on feature-level representations.

**Split dataset:** The dataset was split into training, validation, and test set, with a 0.7/0.2/0.1 split.

### 3.2 Block 2: transformer-based image segmentation

We adopted and implemented the transformer-based segmentation architecture, called SegFormer (19), for our LarynxFormer framework. The architecture is based on the vision transformer (23). In the next subsection, we explain how the transformer-based segmentation of the LarynxFormer works, the training process of our methods, and how the models are evaluated.

#### 3.2.1 Model architecture

**Figure 5:**
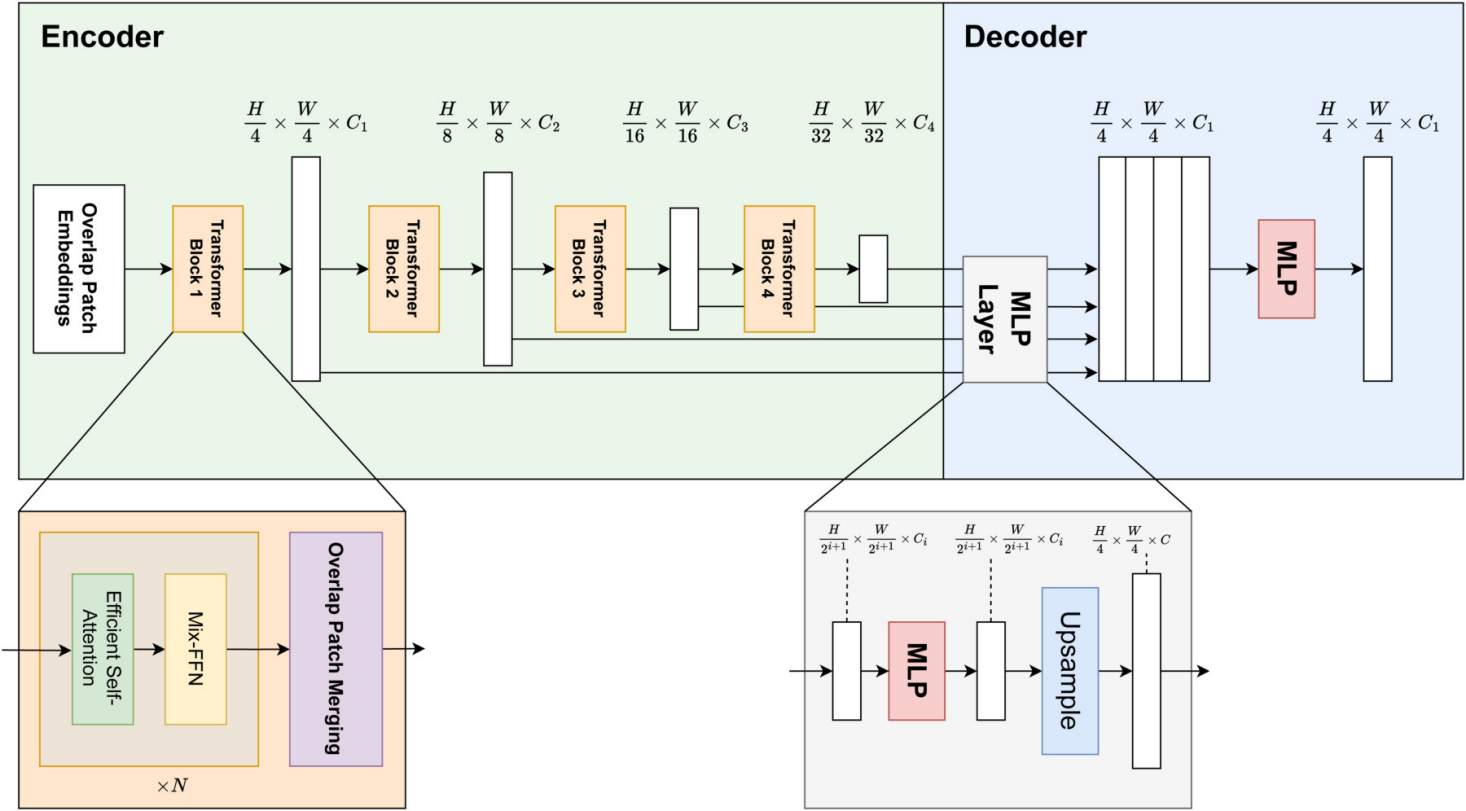
LarynxFormer transformer-based segmentation architecture. Describes the structure of the encoder and decoder. The encoder is responsible for feature extraction, while the decoder performs the segmentation mask prediction (19). *H*, height; *W*, width; *C*, channels; *MLP*, multi-layer perceptron; *Mix-FFN*, mix feed-forward network.

The segmentation block of the LarynxFormer is a simple and efficient architecture. The architecture (see Figure 5) includes two main components, an encoder and a decoder (19). The encoder generates both high-resolution coarse features and low-resolution fine features. These CNN-like features often boost segmentation performance. These features are combined in the decoder using a lightweight MLP decoder to produce the final segmentation.

The model takes an input image of size H×W×3. Using a convolution operation, the overlap patch embedding module splits the image into overlapping patches. The overlapping strategy ensures the sharing of spatial details between patches. Each patch consists of 4×4 pixels. Opting for the smaller patches is beneficial for the dense prediction task. These patches then serve as inputs to the transformer encoder. The encoder consists of 4 transformer blocks, each generating multi-level features with sizes 1/4, 1/8, 1/16, and 1/32 of the original image resolution.

Following this, self-attention is performed, a mechanism for capturing spatial relationships and contextual information from image patches. The sequence K, originally with shape N×C, is first reshaped to a lower-resolution form using a reduction ratio R. Specifically, it is transformed into a tensor $\hat{K}$ of shape $\frac{N}{R}\times (C\cdot R)$ (Equation 1):

(1) $$\hat{K}=\text{Reshape}\;\left(\frac{N}{R},C\cdot R\right)(K)$$

A linear transformation is then applied (Equation 2):

(2) $$K=\text{Linear}\;(C\cdot R,C)(\hat{K})$$

LarynxFormer uses a Mix feed-forward-network (Mix-FFN) module with a 3×3 Conv layer, adequate for offering positional information similar to what positional encoding does. The Mix-FFN module combines a convolutional layer with a feed-forward network. It processes the input feature $x_{\text{in}}$, which comes from the self-attention module, as follows (Equation 3):

(3) $$x_{\text{out}}=\text{MLP}\;(\text{GELU}\;({\text{Conv}}_{3\times 3}\;(\text{MLP}(x_{\text{in}}))))+x_{\text{in}}$$

Here, a 3×3 convolution is used between two MLP layers, and a GELU activation is applied in between. The final output is obtained through a residual connection that adds the input $x_{\text{in}}$ back to the transformed features.

After being downsampled, each output from the transformer blocks is sent to the decoder part of the architecture. Here, the Multilayer perceptron (MLP) layer manages the upsampling and concatenation of features. It uses these fused features to predict a segmentation mask for the input image.

Overall, this architecture’s blend of transformer and CNN features, strategic use of patch size, and a simplified decoder design culminate in a highly effective and adaptive model for dense prediction tasks.

#### 3.2.2 Model training

We opted to train four models for image segmentation. FCN with a ResNet backbone was selected, drawing inspiration from Lin et al.’s success in utilizing an FCN model for larynx segmentation. ResNet (24) is a well-known architecture used for computer vision tasks with the ability to train deep neural networks effectively. Moreover, Mask R-CNN was chosen as the second model. Mask R-CNN is a relatively complex instance segmentation model. For our setup, the two detected objects, for each class, with the highest confidence were selected (trachea and supraglottis) and used as the final segmentation for calculating loss and other metrics, such as dice, IoU, and F1. The SegFormer (19), implemented in the LarynxFormer, was also added, as vision transformers are yet to be tested on laryngeal image segmentation. Transformers have previously been shown to achieve great results within the field of medical image segmentation (25). Finally, an implementation of the U-Net architecture (26), similar to the approach employed by Kruse et al. in GlottisNetV2, was added to the model list.

All models were trained on a Nvidia RTX A1000 6GB graphics card using Python 3.10.13 and PyTorch 2.1.1. AdamW was chosen as the optimizer function due to its fast convergence and weight decay regularization capabilities (27). The learning rate was set to $10^{-4}$ and weight decay to $10^{-2}$. Cross Entropy, widely adopted for image segmentation tasks, was used as the loss function for the training loop. Cross Entropy (CE) (Equation 4) is defined as follows:

(4) $$CE=-\sum_{i}^{C}t_{i}\log\;(f(s{)}_{i})$$

where *C* is the number of classes, $t_{i}$ is the probability of the target class and $f(s{)}_{i}$ is the probability of the predicted output.

The training batch shape was set to Batches×Channels× Height×Width, with a batch size of B=4, C=3 channels (background, trachea and supraglottis), height of H=400 px and width W=500 px.

#### 3.2.3 Evaluation setup

The evaluation of models involves using metrics for direct comparison, which is our primary method for assessing performance. The dice similarity coefficient (Equation 5), a measurement for pixel similarity, is our main metric for evaluating performance. The equation is

(5) $$DSC=\frac{2\times |T\cap P|}{\left|T|+|P\right|}$$

where T is the target pixels and P is the predicted pixels. Also, Intersection over Union (IoU), F1, precision, and recall were all considered supplementary metrics to enhance the comprehension of model performance and strengthen the rationale behind the evaluation.

In a clinical setting, efficiency may be a crucial factor. For instance, Lin et al. focus on real-time segmentation and analysis during the CLE-test (12), which can be a realistic use case for a future segmentation tool. Regardless, a faster model is always advantageous. Thus, we also consider efficiency in terms of model inference time and FPS.

In addition to metrics, visualization of the segmentation masks is essential to gain insight into their actual performance. This visual evaluation includes assessing factors such as the smoothness of mask boundaries, the presence of random noise, and the occurrence of pixel gaps.

The evaluation of the optimal model will prioritize, in order, performance metrics, efficiency, and visualization.

### 3.3 Block 3: post-processing

**Figure 6:**
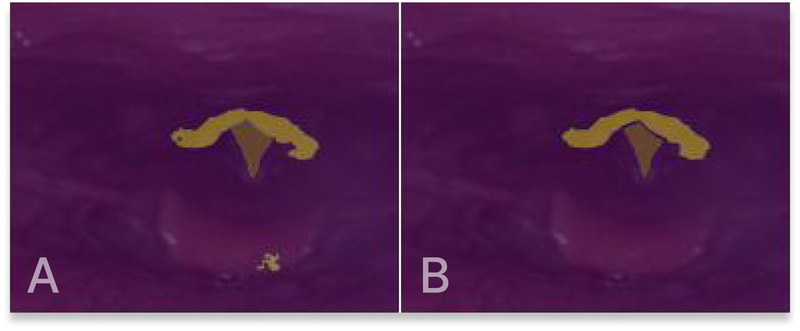
(**A**) Original segmentation mask output. (**B**) Segmentation mask after post-processing.

As a last step, the visual appearance of the generated masks is refined through a sequence of post-processing steps, implemented using OpenCV. Three steps have been assembled, starting with removing outliers. Outliers, which may be present in particular images, are addressed by removing structures that deviate from the largest structure, as they primarily contribute to unwanted noise. Subsequently, evident gaps within the segmentation masks are eliminated, and the boundaries of the masks are smoothed. Figure 6 shows an example of the post-processing of a segmentation mask.

## 4 Results and discussion

This section presents, compares, and discusses the results, including our framework, each model’s metrics, efficiency, and overall performance.

### 4.1 LarynxFormer framework

As an important foundation for the model training, we present a framework for preprocessing CLE-test data for ML purposes. Our dataset and pipeline are designed for use with data from the Bergen ILO Group, but can also be adapted for other clinics. The framework pipeline is an end-to-end solution for data preparation, including both fully automatic and manual steps. The implementation details of the framework can be found on GitHub.^1^ The most time-consuming step is the manual labeling of laryngeal images. We’ve already labeled a good amount of images for this project, but future additions are welcome. The framework lays a base for the group’s future research within the field of ML and EILO.

### 4.2 Model performance

Four models were trained on the same pre-processed dataset. The performance metrics for both trachea and supraglottis and inference times for each model are shown in Table 1. The dice similarity coefficient is our chosen metric for evaluating performance numerically. Inference time is also included in the comparison of the models. In clinical settings, where efficiency may be crucial, inference time is vital in potential future EILO diagnostic tools.

**Table 1:** Evaluation metrics and inference time for each model.

Model	Trachea						Supraglottis			Inference time		
	Dice	IoU^a^	F1	Prec.^b^	Recall	Dice	IoU^a^	F1	Prec.^b^	Recall	Duration	FPS^c^
FCN w/ResNet	0.929	0.850	0.732	0.620	0.908	0.831	0.699	0.666	0.567	**0.833**	59.0 ms	17
Mask R-CNN	0.934	**0.877**	**0.846**	**0.865**	0.839	0.805	0.673	**0.751**	**0.791**	0.727	107.6 ms	9
U-Net	**0.936**	0.858	0.714	0.619	0.855	0.790	0.637	0.616	0.598	0.643	60.9 ms	16
LarynxFormer	0.935	0.864	0.753	0.647	**0.912**	**0.834**	**0.702**	0.675	0.594	0.807	**52.7** ms	**19**

The table presents the performance and efficiency for each model. For dice, IoU, F1, Prec., recall, and FPS, higher scores are better. For duration, lower is better. The best scores are in bold font.

^a^Intersection over Union.

^b^Precision.

^c^Frames per second.

Looking at the numbers in the table, LarynxFormer gives the most promising results. Its dice score shows great results for both the trachea and supraglottis structure (0.935 and 0.834), reaching the highest dice score for supraglottis, 5.6% better than U-Net. LarynxFormer also has the quickest inference time of 52.7 ms or 19 FPS. Mask R-CNN’s results also look promising, outscoring LarynxFormer in several performance metrics, such as IoU, F1, and precision. However, the average inference time for Mask R-CNN is more than doubled compared to LarynxFormer, with LarynxFormer demonstrating over a 100% increase in FPS. Given these two models’ comparable scoring performance metrics, the inference time is the deciding factor in determining the LarynxFormer as the optimal model.

**Figure 7:**
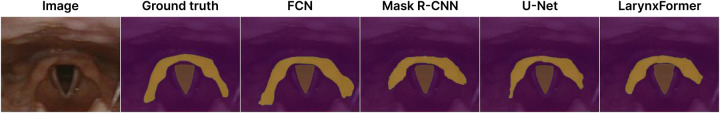
Segmentation masks. Dice scores trachea and supraglottis—FCN: 0.95/0.84, Mask R-CNN: 0.97/0.78, LarynxFormer: 0.97/0.83, U-Net: 0.95/0.83.

The FCN model with ResNet backbone consistently performs well on most metrics, being close to LarynxFormer. Also, the model’s inference time of 17 FPS is almost as good as LarynxFormer’s 19 FPS. The visualization of the FCN masks showed that it has the most smooth edges, even before the post-processing (Figure 7). U-Net’s trachea segmentation results are great, achieving the best dice score of 0.936. However, its supraglottis segmentation performance is less impressive, scoring lowest on several metrics with 0.790. Given the overall performance across classes, we find that Mask R-CNN and LarynxFormer provide more reliable results than U-Net.

Mask R-CNN is by far the best model in terms of the precision metric, scoring 0.865 for the trachea and 0.791 for the supraglottis. Upon reviewing the segmentation masks from the test results, the correlation between the higher precision scores and Mask R-CNN’s performance becomes apparent, as it rarely misclassifies pixels outside the target area. This might occur due to its region of interest layer, which crops the designated area for each class as one of the initial model steps. The segmentation masks produced by Mask R-CNN are also the most visually appealing due to the model’s high precision. In situations where inference time is not critical, Mask R-CNN would be a preferred pick.

All models trained in this study, including the best-performing LarynxFormer, are not ready for use in a clinical setting. Many prediction mistakes are still made, and a bigger dataset and more computational power are needed to train more robust models. Moreover, the segmentation masks can be further analyzed to provide more useful insights in addition to the position of the vocal folds and supraglottis. This research primarily focuses on comparing the models to identify the most suitable solution for using ML to segment the laryngeal structures.

### 4.3 Training process

**Figure 8:**
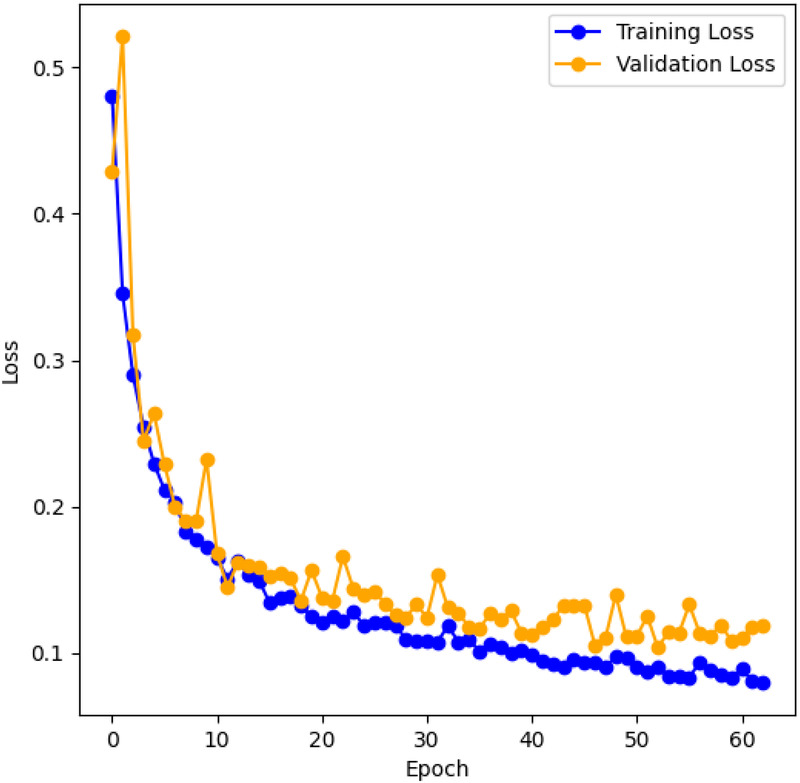
LarynxFormer training and validation loss. The graph shows the training and validation cross-entropy loss during training for each training epoch.

Figure 8 shows the training and validation cross-entropy loss for the LarynxFormer training procedure. The training lasted for 2 h across 63 epochs. Early stopping was implemented to prevent overfitting, allowing a maximum of 10 epochs without increasing validation loss. After testing, 10 epochs for early stopping turned out to be appropriate. Using less than 10 epochs resulted in the model stopping too early, with a small relative decrease in loss. Values higher than 10 led to overfitting of the model. Consequently, the final model version was selected after 53 epochs of training. The hyperparameters for each of the models can be found in Table 2.

**Table 2:** Hyperparameters for each model.

Model	Batch size	Optimizer	Learning rate	Loss function
SegFormer	4	AdamW	$1\times 10^{-4}$	CrossEntropy
Mask R-CNN	2	AdamW	$1\times 10^{-4}$	CrossEntropy
U-Net	4	AdamW	$1\times 10^{-4}$	CrossEntropy
FCN ResNet50	4	AdamW	$1\times 10^{-4}$	CrossEntropy

It’s important to note that comparing the training durations of the models is challenging since some models surpassed GPU memory capacity, necessitating the use of slower CPU memory, which substantially extended the training time.

### 4.4 Practical challenges

Accurate segmentation of the CLE test images poses several challenges that must be addressed to make a functional diagnostics tool for EILO. Our models encounter difficulties in certain areas, suggesting they are imperfect. Differences in lighting, especially in darker conditions, pose challenges, as do unfamiliar angles. Random rotation is integrated into the data augmentation pipeline to enhance the handling of various angles. Improving our ability to tackle these challenges depends on having a larger dataset. Unfortunately, the 6 GB GPU memory limit prevented us from expanding our data further. It’s advisable to invest in higher memory capacity for future studies.

The segmentation mask boundaries, especially for the larger supraglottis structure, frequently lack smoothness and display rough edges. While the FCN model handles this challenge well, the problem is more significant for the remaining models. Boundary smoothing is applied to the segmentation masks as a post-processing step to compensate for the jagged edges. Moreover, a tool for filling segmentation gaps and removing small outliers is also applied to the masks, improving their visual look.

Another notable challenge is the manual process of selecting frames and labeling masks. Like all manual tasks that require human involvement, these steps are prone to bias. Domain experts may assign different labels to the masks depending on their expertise and interpretations. This aspect should always be considered when developing such models, especially in medicine. Labeling the supraglottis correctly, in particular, can pose challenges due to the difficulty in distinguishing its exact boundaries.

### 4.5 Ablation study

An ablation study was performed to evaluate the contribution of the data augmentation steps in the pre-processing block.

Table 3 shows the evaluation metrics for LarynxFormer trained on a dataset with and without data augmentation. For all metrics in the table, higher values are better. The results clearly show that the pre-processing steps make a difference in the model performance. All metrics show a significant improvement when trained with the data augmentation step, with a maximum improvement of 19.4% (from 0.676 to 0.807) for supraglottis recall. The model trained without augmentation has a larger relative ratio between the training and validation loss compared to the model trained with augmentation, indicating a more generalized model when training with data augmentation. This makes sense, considering that data augmentation is often applied with the aim of improving model generalization. The ablation study further indicates that our post-processing steps do not affect the model’s performance, primarily serving visual enhancement purposes.

**Table 3:** Evaluation metrics for LarynxFormer trained on a dataset with and without data augmentation.

Model performance	Trachea						Supraglottis			
	Dice	IoU^a^	F1	Prec.^b^	Recall	Dice	IoU^a^	F1	Prec.^b^	Recall
Without data augmentation	0.896	0.792	0.694	0.615	0.801	0.781	0.629	0.605	0.562	0.676
With data augmentation	**0.935**	**0.864**	**0.753**	**0.647**	**0.912**	**0.834**	**0.702**	**0.675**	**0.594**	**0.807**
Improvement	4.4%	9.1%	8.5%	5.2%	13.9%	6.8%	11.6%	11.6%	5.7%	19.4%

The table compares the performance of LarynxFormer trained on the dataset with and without data augmentation. The improvement row presents the improvement of training with data augmentation vs. without. Higher values are better.

^a^Intersection over Union.

^b^Precision.

## 5 Conclusions

This study has developed the LarynxFormer, a proposed new framework including pre-processing, transformer-based segmentation and post-processing pipeline. Our findings indicate that the framework’s transformer-based segmentation outperforms the prior state-of-the-art techniques, fully convolutional network (FCN), Mask R-CNN, and U-Net for laryngeal segmentation, in terms of performance and efficiency. Considering these results, we conclude that this study contributes to the advancement of objective ML-based diagnostic tools for EILO. Future work could enhance the model performance using a larger dataset, add more computational resources to build a more robust model, experiment more with the architectural details of the transformer model, and potentially use pre-trained model weights. Further, with a more robust segmentation model, classification and CLE-scoring using ML are interesting topics for investigation. Moreover, collaboratively training models on distributed datasets from various medical institutions could facilitate the development of more robust and generalizable segmentation models.

## Data Availability

The original contributions presented in the study are included in the article, further inquiries can be directed to the corresponding author/s.

## Footnotes

^1^ https://github.com/Ci2Lab/Rune_LarynxFormer_Framework
